# Tooth extractions in Orthodontics: first or second premolars?

**DOI:** 10.1590/2177-6709.24.3.088-098.bbo

**Published:** 2019

**Authors:** Telma Martins de Araújo, Luciana Duarte Caldas

**Affiliations:** 1 Universidade Federal da Bahia, Faculdade de Odontologia, Departamento de Odontologia Social (Salvador/BA, Brazil).; 2 Universidade Federal do Rio de Janeiro, Programa de Pós-Graduação em Odontologia (Rio de Janeiro/RJ, Brazil).

**Keywords:** Malocclusion, Angle Class I malocclusion, Tooth extraction

## Abstract

Tooth crowding and protrusions demand rigorous attention during orthodontic planning that includes the extraction of first and second premolars. Some characteristics, such as dentoalveolar bone discrepancies, maxillomandibular relations, facial profile, skeletal maturation, dental asymmetries and patient cooperation, are important elements of an orthodontic diagnosis. This study discusses the options of treatments with extractions and describes the correction of a Class I malocclusion, bimaxillary protrusion, severe anterior crowding in both dental arches and tooth-size discrepancy, using first premolar extractions.

## RESUMO

Apinhamentos e protrusões dentárias têm requerido rigor durante o planejamento ortodôntico envolvendo exodontias de primeiros e segundos pré-molares. Algumas características como discrepância entre dentes e suas respectivas bases ósseas, relacionamento maxilomandibular, perfil facial, maturação esquelética, assimetrias dentárias, patologias e cooperação do paciente têm sido consideradas elementos importantes de diagnóstico ortodôntico. Sendo assim, o presente artigo teve por objetivo discutir as opções de tratamento com extrações dentárias e apresentar a correção de uma má oclusão de Classe I, biprotrusão, com severo apinhamento anterior em ambas as arcadas e discrepância de tamanho dentário, por meio da exodontia de primeiros pré-molares.

## INTRODUCTION

The correct diagnosis of orthodontic cases of Angle Class I malocclusion that require the extraction of the first or second premolars is not always an easy task, especially in a large group known as borderline cases.^1^

Some characteristics, considered important elements of a diagnosis, should be rigorously assessed during planning when it includes extraction of first and second premolars. These characteristics are: discrepancy between teeth and alveolar bone, maxillomandibular relationships, facial profile and facial pattern, skeletal maturation, tooth asymmetries, diseases and patient cooperation.^2^ However, in some cases, a single characteristic may alone define whether the first or the second premolars should be extracted. 

As part of this analysis, tooth crowding, one of the most frequent components of Angle Class I malocclusion, may aggravate with occlusal maturation^3^ and become one of the main aesthetic complaints of patients that seek orthodontic treatment.^1^

Tooth-size discrepancy may also be associated with crowding and substantially accentuate malocclusion.^4^ In the 1950s, Bolton^5^ ^,^ ^6^ established ideal proportions to determine the adequate harmony between maxillary and mandibular teeth. Cases with tooth-size discrepancies may require interproximal stripping, reshaping and even extractions.^7^

One of the first orthodontists to indicate permanent tooth extractions to correct malocclusions was Charles Tweed, who found that only 20% of his clinical cases treated without extractions were successful.^8^ However, his ideas were considerably different from the non-extractionist theory supported by his professor, Edward Angle. Today, premolar extractions are well accepted in the treatment of cases of malocclusion that include severe crowding, unilateral agenesis, bimaxillary protrusion, convex facial profiles and large cephalometric discrepancies, as well as in borderline cases.^2^

Proffit and Fields^9^ created a guide of contemporary procedures to evaluate the need of extractions in cases of Class I malocclusion with crowding or protrusion. The first premolars are usually the teeth chosen because of their position and compatible size with most types of discrepancies in cases that require the retraction of anterior teeth. As a rule, the extraction of second premolars is not indicated for cases with great discrepancies.^2^

 Some studies evaluated the impact of first premolar extractions on the lips and found that, for each 1 mm of maxillary incisor retraction, upper lip mean retraction is 0.75 mm,^10^ 0.64 mm^11^ or only 0.5 mm.^12^ For the lower lip, each 1 mm of mandibular incisor retraction corresponded to a mean retraction of 0.6 mm^13^ or 0.78 mm.^12^ Therefore, space closure by retraction of anterior teeth tends to have a much greater impact on facial profile than second premolar extractions. The present study describes the correction of a Class I malocclusion, bimaxillary protrusion, severe anterior crowding in both dental arches and tooth-size discrepancy, using first premolar extractions.

## CASE REPORT

**Figure 1:**
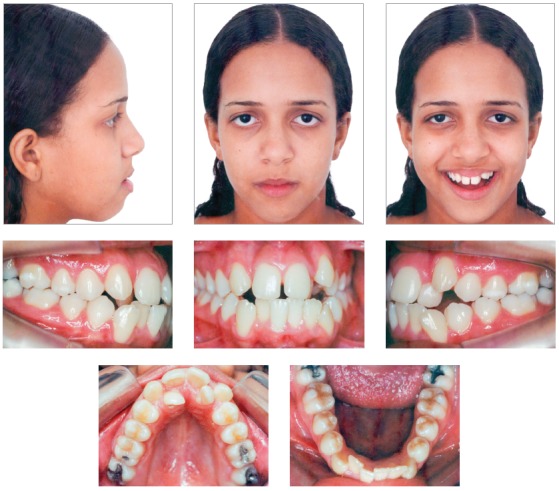
Initial facial and intraoral photographs.

A 14-year 8-month-old brown-skinned female patient sought dental care at the Department of Orthodontics of the Federal University of Bahia, for corrective orthodontic treatment. Facial analysis revealed no passive lip sealing, eversion of lower lip, decreased nasolabial angle, dolichofacial pattern, augmented lower third of the face, and deficient malar and paranasal regions, which was confirmed by the exposure of the outermost layer of the ocular globe (sclera) on the frontal photograph (Fig 1). The patient had good general health, regular oral hygiene, moderate frequency of carious lesions, satisfactory dental care and normal periodontium.

**Figure 2:**
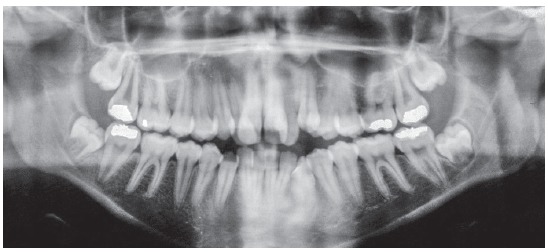
Initial panoramic radiograph.

**Figure 3:**
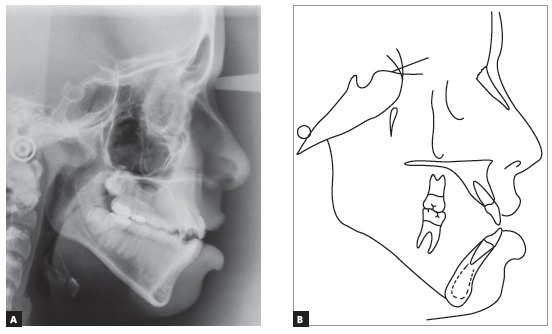
Initial lateral cephalometric radiograph and tracing.

The baseline panoramic radiograph showed developing third molars (Nolla’s stages 6 to 7), with a mesial angulation and overlying images of the anterior region, due to severe tooth crowding. The other teeth and bone structures were normal (Fig 2). The lateral cephalometric radiograph showed skeletal Class I relationship according to ANB angle (2^o^), but Class III maxillomandibular relationship according to Wits (-2 mm) (SN-GoGn = 40^o^, FMA = 36^o^, Y axis = 68^o^, facial angle=84^o^). Dental pattern analysis revealed proclined and protruded incisors, especially lower incisors (1.1 = 110^o^, 1.NA = 29^o^, 1-NA = 8 mm, 1.NB = 36^o^, 1-NB = 11 mm, IMPA = 96^o^) (Fig 3; Table 1, column A).

**Table 1:** Cephalometric measurements at baseline (A), final (B) and nine years follow-up.

	Measures		Normal	A	B	C	A/B diff.
Skeletal pattern	SNA	(Steiner)	82°	81°	82°	82°	1
	SNB	(Steiner)	80°	79°	80°	80°	1
	ANB	(Steiner)	2°	2°	2°	2°	0
	Wits	(Jacobson)	♀ 0 ± 2 mm ♂ 1 ± 2 mm	-2mm	0mm	0mm	2
	Angle of convexity	(Downs)	0°	8°	5°	5°	3
	Y-axis	(Downs)	59°	68°	65°	65°	3
	Facial angle	(Downs)	87°	84°	83°	83°	1
	SN-GoGn	(Steiner)	32°	40°	37°	37°	3
	FMA	(Tweed)	25°	36°	33°	33°	3
Dental pattern	IMPA	(Tweed)	90°	96°	87°	87°	9
	1.NA (degrees)	(Steiner)	22°	29°	27°	27°	2
	1-NA (mm)	(Steiner)	4 mm	8mm	6mm	6mm	2
	1.NB (degrees)	(Steiner)	25°	36°	23°	23°	13
	1-NB (mm)	(Steiner)	4 mm	11mm	6mm	6mm	5
	- Interincisal angle	(Downs)	130°	110°	126°	126°	16
Profile	Upper lip - S-line	(Steiner)	0 mm	0mm	-1,5mm	-1,5mm	1,5
	Lower lip - S-line	(Steiner)	0 mm	7mm	1,5mm	1,5mm	5,5

The evaluation of cervical vertebrae^14^ on the lateral radiograph revealed that C3 was square and had a concave inferior border, a sign of pubertal growth spurt or post-spurt development (Fig 3). 

Intraoral clinical examination revealed Angle Class I malocclusion, bimaxillary protrusion, slight maxillary transverse deficiency, severe anterior crowding in both dental arches, discrepancy of -5.5 mm in the mandible and -11.2 mm in the maxilla, associated with anteroinferior Bolton discrepancy of +2.8 mm. Incisors had an edge-to-edge relation; teeth #12, #22 and #42 were retroclined. Upper midline was 2 mm to the right, and lower midline, 1 mm to the left (Fig 1). Treatment objectives were: correct tooth size discrepancy in the anteroinferior segment; eliminate crowding to make oral hygiene easier; improve the shape of both arches and establish proper overjet and overbite; achieve harmonious facial profile and lip position. For that purpose, an orthodontic setup model was projected to test the treatment plan, which included the extraction of the four first premolars (Fig 3). The plan included intraoral appliances for anchorage: a Nance button for the maxilla, and a lingual arch for the mandible. 

### Treatment options

Two treatment plans to achieve normal occlusion and improve facial profile were presented, with and without orthognathic surgery. Initially, maxillary advancement was suggested because of the patient’s malar and paranasal deficiency. However, although maxillary advancement by means of orthognathic surgery has been classified as an efficient form of treatment,^16^ the patient’s guardians did not give permission for the surgery, and, in addition, the patient did not have any complaints about her facial appearance. Her choice, therefore, was to conduct only the corrective orthodontic treatment with extractions. The first premolars were selected because the space created in both dental arches would allow for the retraction of the anterior teeth, correcting malocclusion and improving her facial profile. 

### Treatment progression

A standard 0.022 x 0.028-in Edgewise fixed appliance (Morelli, Sorocaba, Brazil) was used, and all molars received bands. After the maxillary Nance button and the mandibular lingual arch were placed, brackets were bonded to the canines and second molars.

After the first premolars were extracted, canine retraction was initiated, using stainless steel 0.018 x 0.025-in segmental archwires with T-loops (Morelli, Sorocaba, Brazil). After that, brackets were bonded to the incisors, to start leveling. A removable plate with posterior biteplate, combined with a coil spring on the lingual surface of tooth # 12, was used for disocclusion and for allowing single tooth crossbite correction. Then, a sequence of stainless steel 0.014-in to 0.020-in archwires (Morelli, Sorocaba, Brazil) was used for leveling and alignment, and canine retraction continued using elastic chains (American Orthodontics, Sheboygan, WI).

When canine retraction was completed, the auxiliary intraoral anchorage appliances were removed, and incisor retraction continued with 0.019 x 0.026-in stainless steel teardrop loops (Morelli, Sorocaba, Brazil) placed between lateral incisors and canines. Interproximal stripping of anterior mandibular teeth eliminated the baseline tooth-size discrepancy (+2.8 mm) in this region. 

At the finishing stage, special attention was paid to the coordination of maxillary and mandibular archwires, and to the ideal torque for all teeth, so that proper dental function and aesthetics were achieved.

## RESULTS

The planned orthodontic outcomes were achieved at the end of the active treatment phase. For retention of the mandible, a stainless steel 0.028-in lingual arch (Morelli, Sorocaba, Brazil) was bonded to the canines, and segments of 0.020-in twist flex wire (Morelli, Sorocaba, Brazil) were bonded to teeth #33-35 and #43-45, to prevent the creation of diastemas in the extraction spaces. For the maxilla, a removable wraparound retainer was manufactured with a 0.032-in stainless steel wire (Morelli, Sorocaba, SP, Brazil), which the patient should wear for 20 hours a day for three months, 12 hours a day for six months, and at night only after that. 

After treatment, extraoral clinical examination revealed a more harmonious face, substantial improvement of the facial profile resulting from premolar extractions and effective vertical control, and good passive lip seal due to retraction of mandibular incisors (Fig 5). The dental examination revealed that orthodontic treatment resulted in: normal occlusion, with molar relationship preservation and canine relationship; normal overjet and overbite; and corrected midline (Fig 5), as planned in the orthodontic setup model (Fig 3).

**Figure 4:**
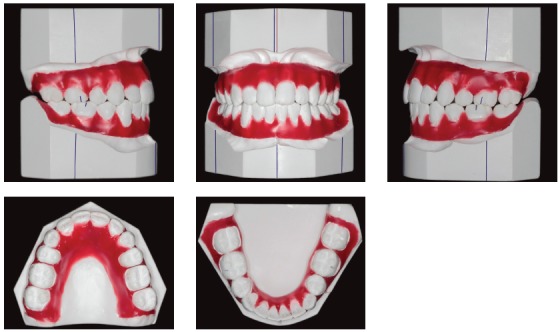
Setup and simulation of planned treatment.

**Figure 5:**
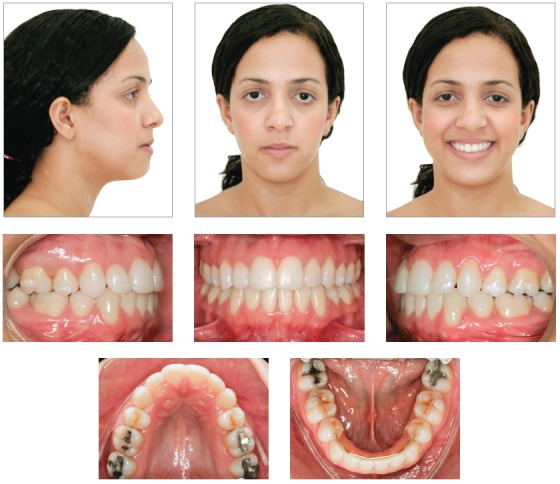
Facial and intraoral photographs after treatment.

**Figure 6:**
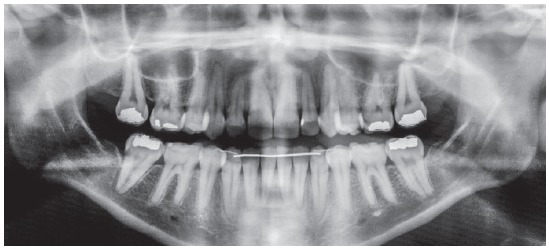
Panoramic radiograph after treatment.

In the final examinations, the panoramic radiograph showed good parallel relations and root integrity, and the third molars were extracted (Fig 6). The lateral cephalometric radiograph showed that the anteroposterior maxillomandibular relationships kept their balance (ANB = 2^o^, Wits = 0 mm), and the decrease of SN-GoGn (from 40^o^ to 37^o^) and FMA (from 36^o^ to 33^o^) angles indicated that there was good vertical control of orthodontic mechanics during treatment. Dental examinations revealed mandibular incisor retraction and uprighting, as well as a decrease of the 1.NB angle (from 36^o^ to 23^o^). Figures 8 to 10 show that orthodontic results remained stable nine years after treatment.

**Figure 7:**
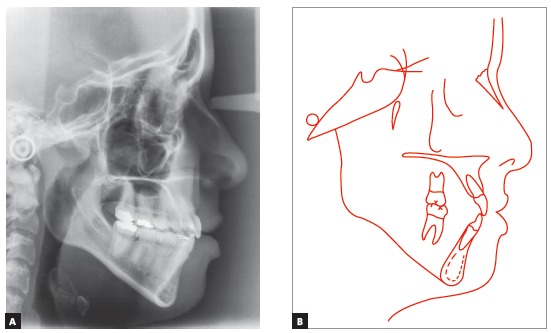
Lateral cephalometric radiograph and tracing after treatment.

**Figure 8:**
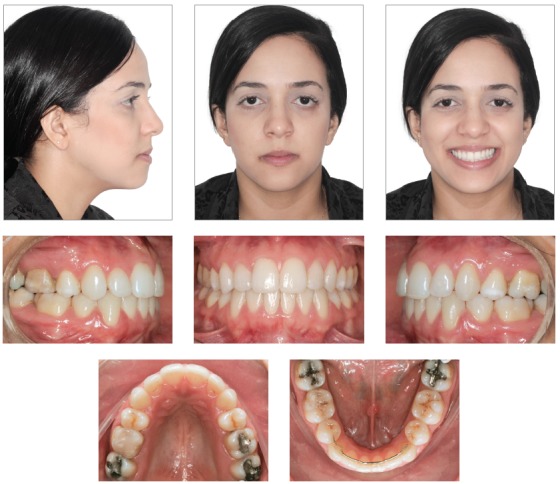
Stability of achieved results, nine years after treatment completion.

**Figure 9:**
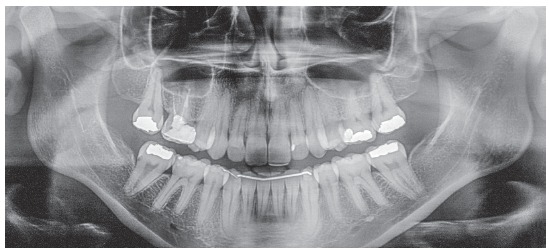
Panoramic radiograph nine years after treatment completion.

**Figure 10:**
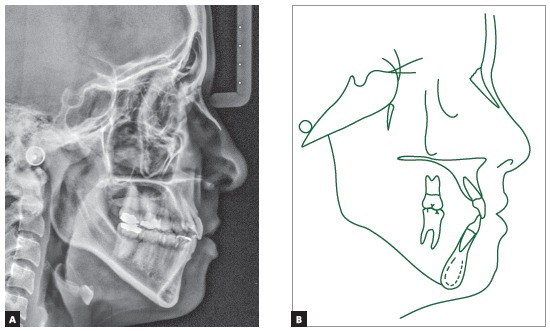
Lateral cephalometric radiograph and tracing nine years after treatment completion.

## DISCUSSION

The high prevalence of tooth crowding poses a constant dilemma to orthodontists: which treatment option to follow, with or without extractions? How many and which teeth should be extracted? Based on studies about different types of treatment to alleviate crowding,^17^ ^-^ ^19^ one of the routine procedures for Class I malocclusion and bimaxillary protrusion is the extraction of the first premolars.^2^ These teeth are usually chosen because of their position and size, which are compatible with most types of discrepancies in cases that require the retraction of anterior teeth.^20^ However, tooth-size discrepancies may also be found in the same case, which will require not only extractions, but also interproximal stripping.^3^ In the case described here, the patient had a tooth-size discrepancy of -5.5 mm in the mandibular arch and -11.2 mm in the maxillary arch, combined with an anteroinferior Bolton discrepancy of +2.8 mm. Therefore, because of the severe crowding in the anterior region in both dental arches, the treatment plan included the extraction of the four first premolars and the interproximal stripping of anterior mandibular teeth to eliminate discrepancies.

**Figure 11:**
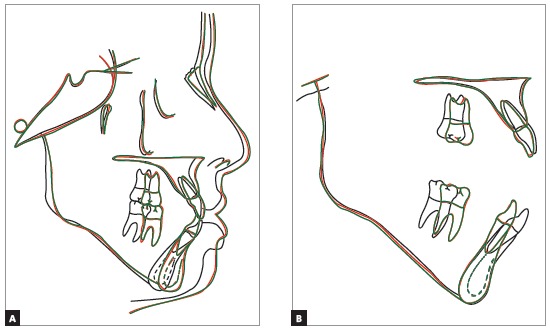
Total (SN line registered at sella) and partial superimpositions.

Several anchorage techniques are used for the retraction of anterior teeth in treatments that include extraction of the first premolars.^21^ Skeletal anchorage devices, such as mini-implants and miniplates, have been widely used for this purpose because of the comfort and aesthetic improvement that they provide.^22^ ^-^ ^24^ For this clinical case, intraoral appliances and a Nance button with lingual arch were chosen. The analysis of total and partial superimpositions confirmed the efficacious result of the adequate use of these appliances, with effective control of vertical growth and slight horizontal maxillary and mandibular growth (Table 1, column C), as well as mild extrusion and mesial migration of molars, followed by incisor retraction (Fig 11).

Miyake, Ryu and Himuro^25^ compared the dental arch form at baseline and after the extraction of premolars in individuals with Class I crowding treated with preadjusted brackets. They found that the maxillary dental arch might become tapered after treatment with extractions. In this clinical case, brackets without a prescription were used, and all archwires were made of stainless steel. Therefore, it was possible to accurately follow the original form of the dental arches, resulting in well-coordinated arches with an adequate form, which explains its excellent stability nine years postretention (Fig 8). 

A factor routinely investigated in recent years is the relationship between lips and soft profile, as well as the vertical changes after treatment with premolar extractions.^17^ ^,^ ^26^ ^-^ ^29^ Regardless of the method used for two-dimensional^17,26,28^ or three-dimensional^29^ evaluations, lip protrusion substantially improves after the extraction of these teeth. Leonardi et al^27^ conducted a systematic review and found that the upper lip retracts a mean of 2 - 3.2 mm and the lower lip, 2 - 4.5 mm, while the nasolabial angle increases. These changes are significant, especially in light-skinned individuals.^26,28^ The analysis of final face photographs and superimpositions (Figs 5 and 11) showed passive lip sealing and significant improvement of profile and facial harmony. This resulted from the effective vertical control and proper positioning of incisors on the alveolar bone, which was preserved due to the correct and recommended use of a removable wraparound maxillary retainer and a mandibular lingual arch bonded to the canines (Fig 8).

Lastly, the other treatment alternative suggested for this clinical case would be maxillary advancement by means of orthognathic surgery. Moragas et al^16^ conducted a systematic review of studies about changes in soft and hard tissues using maxillary repositioning in orthognathic surgeries. They found that, although there are other publications about these changes, more prospective studies have to be conducted to stratify some factors, such as type of osteotomy technique, magnitude of the movement, age, sex, ethnicity, and quantity and quality of soft tissues. Despite the nasal and paranasal deficiency diagnosed, this patient did not report any dissatisfaction with her face, and her guardians did not accept a surgical approach. However, the fact that the maxillary advancement using orthognathic surgery would provide a better facial profile was recorded in the case’s file. 

## FINAL CONSIDERATIONS

The correction of severe crowding in this case of Class I malocclusion was successful after extraction of the four first premolars and use of intraoral anchorage. There was significant improvement of dental and gingival margin aesthetics, which gave the patient a quite agreeable and harmonious smile after orthodontic treatment. In addition, treatment established functional movements that ensured this case’s excellent stability nine years postretention. The analysis of the final casts and radiographs, as recommended by the American Board of Orthodontics (BBO),^30^ indicated that this treatment scored 15 points, with good treatment completion. For these reasons, this case was granted the first runner-up award of the Clinical Forum of the Congress of the Brazilian Association of Orthodontics (ABOR).

## References

[1] Liu Z, McGrath C, Hägg U (2009). The impact of malocclusion/orthodontic treatment need on the quality of life A systematic review. Angle Orthod.

[2] Ruellas ACO, Ruellas RMO, Romano FL, Pithon MM, Santos RL (2010). Tooth extraction in orthodontics an evaluation of diagnostic elements. Dental Press J Orthod.

[3] Normando D, Almeida MA, Quintão CC (2013). Dental crowding the role of genetics and tooth wear. Angle Orthod.

[4] Othman S, Harradine N (2007). Tooth size discrepancies in an orthodontic population. Angle Orthod.

[5] Bolton WA (1958). Disharmony in tooth size and its relation to the analysis and treatment of malocclusion. Angle Orthod.

[6] Bolton WA (1962). The clinical application of a tooth-size analysis. Am J Orthod Dentofacial Orthop.

[7] Othman SA, Harradine NW (2006). Tooth-size discrepancy and Bolton's ratios a literature review. J Orthod.

[8] Tweed CH (1945). Indications for the extraction of teeth in orthodontic procedure. Am J Orthod Oral Surg. 1944-.

[9] Proffit WR, Fields JRW (1995). Ortodontia contemporânea.

[10] Ramos AL, Sakima MT, Pinto AS, Bowman J (2005). Upper lip changes correlated to maxillary incisor retraction - a metallic implant study. Angle Orthod.

[11] Talass MF, Tollaae L, Baker RC (1987). Soft-tissue profile changes resulting from retraction of maxillary incisor. Am J Orthod Dentofacial Orthop.

[12] Massahud NV, Totti JIS (2004). Estudo cefalométrico comparativo das alterações no perfil mole facial pré e pós-tratamento ortodôntico com extrações de pré-molares. J Bras Ortodon Ortop Facial.

[13] Kusnoto J, Kusnoto H (2001). The effect of anterior tooth retraction on lip position of orthodontically treated adult Indonesians. Am J Orthod Dentofacial Orthop.

[14] Baccetti T, Franchi L, McNamara JA (2002). An improved version of the cervical vertebral maturation (CVM) method for the assessment of mandibular growth. Angle Orthod.

[15] Araújo de TM, Fonseca LM, Caldas LD, Costa-Pinto RA (2012). Preparation and evaluation of orthodontic setup. Dental Press J Orthod.

[16] San Miguel Moragas J, Van Cauteren W, Mommaerts MY (2016). A systematic review on soft-to-hard tissue ratios in orthognathic surgery part I Maxillary repositioning osteotomy. J Craniomaxillofac Surg.

[17] Marques LS, Chaves KC, Ramos-Jorge ML, Pereira LJ (2011). Extraction of four premolars in Black patients with bi-protrusion: aesthetic perceptions of professionals and lay people. J Orthod.

[18] Zhylich D, Suri S (2011). Mandibular incisor extraction: a systematic review of an uncommon extraction choice in orthodontic treatment. J Orthod.

[19] Francisconi MF, Janson G, Freitas KM, Oliveira RC, Freitas MR, Henriques JF (2014). Overjet, overbite, and anterior crowding relapses in extraction and nonextraction patients, and their correlations. Am J Orthod Dentofacial Orthop.

[20] Redahan S, Lagerström L (2003). Orthodontic treatment outcome: the relationship between anterior dental relations and anterior inter-arch tooth size discrepancy. J Orthod.

[21] Lai EH, Yao CC, Chang JZ, Chen I, Chen YJ (2008). Three-dimensional dental model analysis of treatment outcomes for protrusive maxillary dentition comparison of headgear, miniscrew, and miniplate skeletal anchorage. Am J Orthod Dentofacial Orthop.

[22] Thiruvenkatachari B, Ammayappan P, Kandaswamy R (2008). Comparison of rate of canine retraction with conventional molar anchorage and titanium implant anchorage. Am J Orthod Dentofacial Orthop.

[23] Yao CC, Lai EH, Chang JZ, Chen I, Chen YJ (2008). Comparison of treatment outcomes between skeletal anchorage and extraoral anchorage in adults with maxillary dentoalveolar protrusion. Am J Orthod Dentofacial Orthop.

[24] Li F, Hu HK, Chen JW, Liu ZP, Li GF, He SS (2011). Comparison of anchorage capacity between implant and headgear during anterior segment retraction. Angle Orthod.

[25] Miyake H, Ryu T, Himuro T (2008). Effects on the dental arch form using a preadjusted appliance with premolar extraction in Class I crowding. Angle Orthod.

[26] Hodges A, Rossouw PE, Campbell PM, Boley JC, Alexander RA, Buschang PH (2009). Prediction of lip response to four first premolar extractions in white female adolescents and adults. Angle Orthod.

[27] Leonardi R, Annunziata A, Licciardello V, Barbato E (2010). Soft tissue changes following the extraction of premolars in nongrowing patients with bimaxillary protrusion A systematic review. Angle Orthod.

[28] Konstantonis D (2012). The impact of extraction vs nonextraction treatment on soft tissue changes in Class I borderline malocclusions. Angle Orthod.

[29] Solem RC, Marasco R, Guiterrez-Pulido L, Nielsen I, Kim SH, Nelson G (2013). Three-dimensional soft-tissue and hard-tissue changes in the treatment of bimaxillary protrusion. Am J Orthod Dentofacial Orthop.

[30] Casko JS, Vaden JL, Kokich VG, Damone J, James RD, Cangialosi TJ (1998). Objective grading system for dental casts and panoramic radiographs American Board of Orthodontics. Am J Orthod Dentofacial Orthop.

